# Serum Iodine Levels and 8-Year Survival in Patients After Kidney Cancer Diagnosis

**DOI:** 10.3390/cancers17213400

**Published:** 2025-10-22

**Authors:** Elżbieta Złowocka-Perłowska, Piotr Baszuk, Adam Kiljańczyk, Wojciech Marciniak, Róża Derkacz, Aleksandra Tołoczko-Grabarek, Andrzej Sikorski, Marcin Słojewski, Adam Gołąb, Artur Lemiński, Michał Soczawa, Magdalena Marciniak, Rodney J. Scott, Jacek Gronwald, Jan Lubiński

**Affiliations:** 1 Department of Genetics and Pathology, International Hereditary Cancer Center, Pomeranian Medical University, 70-204 Szczecin, Poland; 2 Read-Gene, Grzepnica, ul. Alabastrowa 8, 72-003 Dobra, Poland; 3 Department of Diagnostic Imaging and Interventional Radiology, Pomeranian Medical University Hospital No 1, 70-252 Szczecin, Poland; 4 Department of Urology and Oncological Urology Clinic, Pomeranian Medical University, 71-899 Szczecin, Poland; 5 Department of Biochemical Research, Pomeranian Medical University, 70-111 Szczecin, Poland; 6 School of Biomedical Sciences and Pharmacy, Centre for Information-Based Medicine, Hunter Medical Research Institute, University of Newcastle, Newcastle, NSW 2305, Australia; 7 Division of Molecular Medicine, Pathology North, NSW Pathology, Newcastle, NSW 2305, Australia

**Keywords:** kidney cancer, survival, iodine

## Abstract

Background/Objectives: The aim of this study was to evaluate the association between serum iodine (I) levels and kidney cancer mortality. The role of serum iodine levels in relation to 8-year survival rates in survivors of kidney cancer has not previously been established. In this prospective study, we analyzed 284 consecutive, unselected survivors of kidney cancer and determined their 8-year survival in relation to iodine levels. Methods: Micronutrient levels were measured using inductively coupled plasma mass spectrometry (ICP-MS). Each survivors of kidney cancer was assigned to one of four groups based on the quartile distribution of iodine levels, ranked in ascending order. The multivariable models included covariates such as age at diagnosis, sex, smoking status, type of surgery, histopathological classification and serum levels of selenium, zinc, copper and the zinc-to-copper ratio. Results: We observed that survivors of kidney cancer with serum iodine levels in quartiles III and IV had significantly higher all-cause mortality compared to those in quartile II (reference quartile) (HR = 2.83; *p* = 0.012; HR = 2.64; *p* = 0.017). Furthermore, multivariable analysis revealed a significant association between serum iodine levels (quartiles III and IV vs. quartile II) and mortality due to kidney cancer progression (HR = 4.17; *p* = 0.031; HR = 3.94; *p* = 0.038, respectively). This association was significant only among men in quartile IV (HR = 16.5; *p* = 0.027). Additionally a positive association was observed between iodine levels in quartile IV and all-cause mortality from non–kidney cancer–related deaths (HR = 5.41; *p* = 0.05). Conclusions: To our knowledge, this study is the first investigation of relationship between serum iodine levels and survival of survivors of kidney cancer.

## Simple Summary

Kidney cancer is among the most common malignancies of the urinary tract. In this prospective study we evaluate the association between serum iodine (I) levels and kidney cancer mortality. We analyzed 284 consecutive, unselected kidney cancer cases and assessed their 8-year survival in relation to I levels. Iodine imbalance, whether due to deficiency or excess, has been associated with adverse physiological effects. Consistent with this, our study demonstrated that serum iodine levels outside the range of 63.42–72.02 µg/L were associated with higher mortality among kidney cancer survivors, mainly driven by progression of the primary malignancy.

## 1. Introduction

Kidney cancer (KC) is one of the most common malignancies of the urinary tract, with an annual world-wide incidence of approximately 431,288 new cases and 179,368 deaths [1]. Men are more likely to develop kidney cancer than women, and the incidence of the disease increases with age [2]. In addition to inherited mutations that influence the risk of kidney cancer, various non-genetic, modifiable factors also play a role. These include obesity, hypertension, smoking, chronic kidney disease, alcohol consumption, and occupational exposure to trichloroethylene and trace elements [3,4].

Heavy metals are naturally occurring elements found in the Earth’s crust. Some of them play essential roles in metabolic processes. Nonetheless, when the concentrations of these essential elements fall below or exceed normal physiological levels, they may become toxic and pose significant health risks. In a previous report, we demonstrated that survivors of kidney cancer with the lowest selenium and zinc levels had significantly higher all-cause mortality compared to those with higher levels of these elements [5]. In a second study, we observed a significantly increased risk of all-cause mortality in survivors of kidney cancer with the highest blood or serum copper concentrations compared to those with lower levels [6]. Research indicates that long-term exposure to metals such as arsenic, cadmium, chromium, copper, iron, lead, mercury, nickel, vanadium and zinc can lead to deleterious health effects in humans, including chronic inflammatory conditions and an increased risk of various cancers, as well as cardiovascular, pulmonary and neurological diseases [7,8,9]. Regarding renal cell carcinoma, heavy metals like cadmium and lead have been identified as potential risk factors [10,11]. Sá et al. detected chromium, iron and copper in renal cancer tissue whereas no such metals were found in adjacent healthy tissue [4]. Various studies have demonstrated an association between iodine deficiency and an increased risk of thyroid cancer, which is among the malignancies most directly influenced by iodine status [12,13,14]. The role of iodine in cancers outside the thyroid suggest that higher blood iodine levels are associated with a significantly lower risk of developing breast cancer [15,16,17,18]. Furthermore, it has been shown that iodine deficiency may be associated with an increased risk of breast, endometrial, ovarian and gastric cancers [16,19,20,21,22,23,24]. There is also some indication of a trend towards a lower prostate cancer risk in men with the highest iodine intake [25,26].

The kidneys, like the thyroid, have the capacity to accumulate and excrete iodine, potentially exposing renal tissues to elevated iodine concentrations [27]. One potential role of iodine in kidney cancer is as a modulator of oxidative stress. Excess iodine can increase the production of reactive oxygen species (ROS), which may cause DNA damage and contribute to carcinogenesis in sensitive tissues. If oxidative stress pathways are activated, this could contribute to tumor initiation or progression. Iodine-induced ROS production, particularly via hydrogen peroxide (H_2_O_2_) has been studied in thyroid and mammary tissues and may be relevant in renal tissues as well. ROS is a well-known promoter of genomic instability and cancer progression [28,29]. Iodine interacts with several trace elements—selenium, zinc, copper, and iron—all of which play essential roles in oxidative stress regulation and immune function. An imbalance in iodine levels may disrupt the homeostasis of metal-dependent antioxidant enzymes, such as glutathione peroxidase, potentially impairing cellular antioxidant defenses [30,31]. The kidneys play a key role in thyroid hormone metabolism. When triiodothyronine (T_3_) undergoes deiodination in the kidneys, the released iodide may be excreted in the urine rather than returning to the general body iodine pool. This process, known as a renal "iodide leak," contributes to iodine presence in renal tissues and urine [32]. Iodide is filtered from the bloodstream through the kidneys and excreted in urine. This process is especially rapid because iodide remains unbound to serum proteins and circulates freely [33]. Studies have linked excessive exposure to iodide to renal dysfunction, transient hypothyroidism, or even tubular necrosis in susceptible populations [34]. The sodium-iodide symporter (NIS, encoded by SLC5A5), responsible for iodine uptake in thyroid cells, has also been identified in non-thyroidal tissues such as the kidney and renal tumors, albeit at low levels. Studies have identified NIS expression in renal tubular epithelium, particularly distal tubule segments [35]. In cancerous tissues, NIS expression may be altered, which could impact iodine accumulation and its downstream biological effects [36,37]. Iodine accumulation in kidney cancer or lesions is most often due to NIS expression—especially in metastatic thyroid cancer cells [38]. Cellular studies have shown that in some cancers (e.g., melanoma, liver, stomach, breast cancer) NIS expression can be pharmacologically induced, e.g., by histone deacetylase inhibitors (HDAC inhibitors) and inhibition of MAPK and PI3K/Akt pathways [39]. Furthermore, some studies suggest altered expression of iodide-handling proteins, such as pendrin (SLC26A4), in renal pathophysiology, including in the context of oncogenesis [40]. In patients with metastatic renal cell carcinoma undergoing treatment with immune checkpoint inhibitors, changes in iodine concentration measured via Dual-Energy CT have been shown to predict treatment response. Patients whose normalized iodine concentration increased from baseline were more likely to respond, even in cases where early size-based imaging did not show tumor shrinkage [41,42]. Although most mechanistic studies of iodine come from thyroid or breast tissue, molecular iodine (I_2_) has been shown to act generally as an antioxidant, differentiator, and immune modulator. It can also form antitumoral derivatives—iodolipids such as 6-iodolactone (6-IL)—which induce apoptosis and inhibit cancer stem-like cells and markers of treatment resistance [23,43].

To the best of our knowledge, there is only one report correlating iodine exposure and survival of cancer patients. Zhang et al. (2022) showed that progression-free survival in gastric cancer patients receiving neoadjuvant chemotherapy is longer with higher levels of iodine [44]. The aim of our study was to evaluate the association between iodine serum levels and kidney cancer mortality.

## 2. Materials and Methods

### 2.1. Study Group

The study cohort comprised 284 consecutive patients diagnosed with kidney cancer at the Clinic of Urology and Oncological Urology, University Hospital in Szczecin, between 2014 and 2017. Written informed consent was obtained from all participants for the collection and analysis of serum samples. Samples were collected at diagnosis, prior to initiation of any therapy, between 8:00 and 11:00 a.m., following at least six hours of fasting. Collected specimens were stored at −80 °C in a dedicated research biobank until analysis. No exclusion criteria were applied based on age, sex, smoking status, surgical approach, histological subtype, or when applicable cause of death. We have no information on environmental or social factors in the patients. A comprehensive summary of the study cohort is provided in Table 1. Vital status and mortality data were obtained from the Polish Ministry of Internal Affairs and Administration in November 2023. The study received ethical approval from the Ethics Committee of Pomeranian Medical University in Szczecin (reference number KB-006/07/22).

### 2.2. Sample Collection and Storage 

A venous blood sample (10 mL) was collected from each participant into a Becton Dickinson Vacutainer tube containing a clot activator (product number 368381, Becton Dickinson, Plymouth, DEV, UK), incubated at room temperature for a minimum of 30 min, and subsequently centrifuged at 1300× *g* for 12 min. The resulting serum was then stored at −80 °C. On the day of analysis, the serum samples were thawed and centrifuged at 5000× *g* for 5 min prior to further processing.

### 2.3. Measurement Methodology

Serum iodine (I) concentration was quantified using inductively coupled plasma mass spectrometry (ICP-MS) with the NexION 350D system (PerkinElmer, Norfolk, VA, USA). Prior to each analytical run, the instrument was calibrated using a series of external standards. Fresh calibration standards for I were prepared daily at concentrations of 1, 2, 3, 4, 5, 10, 50, 75, 100, 120, 150, and 170 µg/L by diluting a 10 µg/mL Multi-Element Calibration Standard 3 (PerkinElmer Pure Plus, Shelton, CT, USA) in blank reagent. Oxygen was used as the reaction gas, and all calibration curves achieved correlation coefficients exceeding 0.999. Serum samples were diluted 40-fold using a blank reagent consisting of high-purity water (>18 MΩ·cm), Triton X-100 (PerkinElmer, Shelton, CT, USA), tetramethylammonium hydroxide (TMAH; Alfa Aesar, Tewksbury, MA, USA), ethylenediaminetetraacetic acid (EDTA; Sigma-Aldrich, St. Louis, MO, USA) and n-butanol (Merck, Darmstadt, Germany). Further methodological details are available upon request. Serum samples were diluted 40-fold using.

### 2.4. Quality Control 

Accuracy and precision of all measurements were verified using certified reference material (CRM), Clincheck Plasmonorm Blood Trace Elements Level 1 (Recipe, Munich, Germany). Additional technical information, such as plasma operating parameters and mass spectrometer acquisition settings, can be provided upon request.

### 2.5. Statistical Analysis 

Cox regression models were used in both univariable and multivariable analyses to examine the relationship between iodine level in serum and kidney cancer survival. Levels of iodine were categorized into four ascending quartiles, with the quartile linked to the lowest number of deaths chosen as the reference for each element. The reference range for serum iodine levels associated with all-cause mortality in kidney cancer survivors is 63.54–71.87 µg/L, corresponding to quartile II. A standardized follow-up period of 8 years was applied in all analyses.

The multivariable models adjusted for multiple factors: age at diagnosis (≤60 vs. >60), sex (female vs. male), smoking status (non-smoker vs. current or former smoker), type of surgery (nephrectomy vs. tumorectomy), Fuhrman Grade (G I–IV), histopathological classification (clear cell vs. papillary–chromophobe) and serum levels of selenium, zinc, copper and the zinc-to-copper ratio. Statistical significance was defined as *p* ≤ 0.05. To visualize survival outcomes across element quartiles, Kaplan–Meier curves were generated.

All statistical analyses were performed using the R programming environment (R version 4.3.2, R Foundation for Statistical Computing, Vienna, Austria, 2023).

## 3. Results

**Figure 1:**
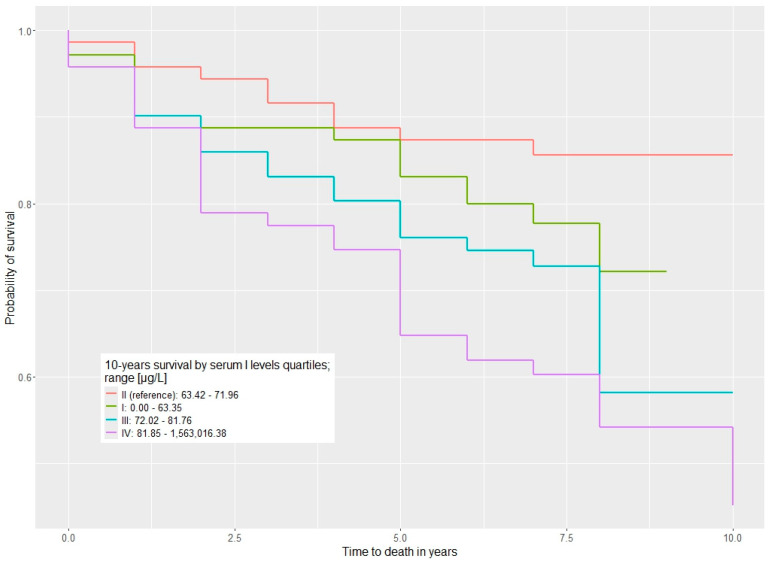
Ten-year overall survival stratified by serum iodine levels (µg/L) across quartiles (Q1–Q4).

The study demonstrated a significant association between serum iodine levels and mortality in survivors of kidney cancer with kidney cancer (Table 2). Survivors of kidney cancer with serum iodine levels ranging from 63.42 to 71.96 µg/L had a significantly better survival rate. In the first quartile (iodine levels up to 63.35 µg/L), there was a non-significant trend toward higher mortality, with a HR of 1.44 (*p* = 0.4). HRs for all-cause mortality were notably elevated for quartile III compared to quartile II (HR = 2.83, *p* = 0.012) and in quartile IV compared to quartile II (HR = 2.64, *p* = 0.017) (Table 2) The survival distributions are visualized with Kaplan–Meier curves in Figure 1.

When stratified by sex, no statistically significant HRs were observed in women in the fourth quartile (HR = 0.57, *p* = 0.4) or in men (HR = 2.63, *p* = 0.09) (Tables S1 and S2, in the Supplementary Material).

A significant relationship was also found between serum iodine levels and mortality due to kidney cancer progression. The HR was 3.94 (*p* = 0.038) for quartile IV vs. II, and 4.17 (*p* = 0.031) for quartile III vs. II (Table 3). This association was particularly strong in men in quartile IV compared to quartile II (HR = 16.5; *p* = 0.027; Table 4), while no significant difference was observed among women (HR = 0.30, *p* = 0.2) (Table S3, in the Supplementary Material).

Additionally, when comparing survivors of kidney cancer in the fourth quartile to those in the third quartile, the HR for mortality due to causes other than kidney cancer was 5.41 (*p* = 0.05) in the overall cohort (Table 5).

## 4. Discussion

The prospective study sample comprised 284 individuals with kidney cancer. We examined 8-year survival of survivors of kidney cancer in relation to serum iodine levels. The best survival was observed in survivors of kidney cancer with serum iodine level 63.42–71.96 µg/L. Individuals with serum iodine levels in quartiles III and IV had significantly higher all-cause mortality compared to those in quartile II. Similarly, the poorest survival related to kidney cancer progression was observed among survivors of kidney cancer in quartiles III and IV compared to quartile II. Notably, these association was evident only in men. Among survivors of kidney cancer who died from non–kidney cancer–related causes, the poorest survival was also observed in those with serum iodine levels in quartile IV. Overall, the relationship between iodine levels and mortality in this population appears to follow a U-shaped pattern. Evidence for a U-shaped association mainly comes from studies on thyroid cancer, where both iodine deficiency and excessive iodine intake are linked to increased risk–whereas moderate intake appears to have a protective effect.

Meta-analyses show that moderate iodine intake (e.g., urinary iodine concentration [UIC] ~100–200 µg/L) is associated with a lower risk of papillary thyroid cancer (PTC), while excessive intake (UIC ≥ 300 µg/L) significantly increases this risk (odds ratio around 4) [45]. At low iodine levels, chronic TSH stimulation leads to persistent thyroid hypertrophy and may ultimately promote tumor development [12]. Excess iodine may increase the expression of cell cycle–related proteins, such as Wee1 and cyclin-dependent kinase 1 in thyroid cancer cells promoting proliferation. The upregulation of these proteins may be part of an aberrant stress response or compensatory mechanism triggered by iodine overload. This pro-proliferative effect appears to be mediated by the PI3K/AKT signaling pathway, a well-known regulator of growth and survival in cancer cells. Pharmacological inhibition of AKT can suppress the expression of Wee1 and CDK1, thereby counteracting the proliferative signal induced by excess iodine and potentially restoring normal cell cycle control [34,46].

Iodine is an essential micronutrient required for the synthesis of thyroid hormones thyroxine (T4) and triiodothyronine (T3). Both insufficient and excessive iodine intake can impair thyroid function and contribute to the development of goiter [47]. Excessive iodine intake is one of the main risk factors for Hashimoto’s thyroiditis, hyperthyroidism, and hypothyroidism [37,48]. High concentrations of iodine in iodinated contrast agents can disrupt thyroid function through the Jod-Basedow effect [49]. Beyond its role in thyroid function, iodine plays a key role in metabolism by enhancing protein synthesis, increasing basal metabolic rate, promoting growth, and maintaining normal brain function [50,51]. Iodine overdose can cause irreversible damage to the retinal pigment epithelium and photoreceptors [52]. Dietary iodine deficiency is associated with intellectual disability, impaired immune response, short stature, delayed physical development, fetal developmental abnormalities, and perinatal mortality [53,54,55,56].

Iodine also exhibits anti-proliferative and pro-apoptotic properties through mitochondrial depolarization and activation of PPARγ receptors, leading to the death of cancer cells [57,58,59]. Iodine deficiency is considered a risk factor for the development of thyroid cancer, especially follicular carcinoma, due to chronically elevated TSH levels and increased cellular proliferation. Conversely, excessive iodine intake may contribute to the development of papillary and anaplastic thyroid cancer by activating the AKT/Wee1/CDK1 signaling pathway, which accelerates the cell cycle and may contribute carcinogenesis [46,60,61,62]. Iodine also inhibits the expression of tumor necrosis factor-α (TNFα) in immune cells, which may help limit inflammatory processes that promote tumorigenesis [63]. Iodine may also have beneficial effects in case of mammary dysplasia and fibrocystic breast disease [64,65]. In breast cancer, iodine has demonstrated promising anti-cancer activity. In MCF-7 and MDA-MB231 cell lines, iodine supplementation led to a reduction in cancer cell proliferation [66]. It also modulates hormone metabolism by upregulating estrogen-metabolizing enzymes such as CYP1A1 and CYP1B1, which may reduce estrogen-driven tumor growth [37]. Additionally, iodine reduced the expression of epithelial–mesenchymal transition markers such as CD44 and vimentin while increasing E-cadherin expression, potentially limiting cellular invasiveness and metastatic potential [67]. Iodine supplementation has also been shown to enhance anti-tumor immune responses by increasing the activity of Th1 lymphocytes, cytotoxic (CD8^+^) T cells, natural killer (NK) cells, and dendritic cells [67].

It has been suggested that iodine levels may influence the survival of patients with gastric cancer. In a study by Zhang et al. involving 85 patients after neoadjuvant chemotherapy with advanced gastric cancer, reduced iodine intake was associated with poorer progression free survival outcomes [44]. Among 71 patients with kidney cancer and serum iodine levels between 63.42–71.96 µg/L (quartile II), survival was significantly better: 10 patients (14%) had died, while 61 (86%) remained alive. In contrast, among 142 patients with iodine levels above 72.02 µg/L (quartiles III and IV), 54 (38%) had died and 88 (62%) were still alive.

Maintaining adequate levels of iodine is essential for overall health, and regular monitoring of iodine status, along with adjusting iodine intake to individual needs, may help reduce the risk of death from various causes. In a study by Maldonado-Araque et al. involving 4370 Spanish adults, individuals with moderate to severe iodine deficiency (urinary iodine concentration < 50 μg/L) had a 71% higher risk of all-cause mortality compared to those with adequate iodine levels (100–300 μg/L) [68]. Similar findings were reported by Inoue et al., who analyzed data from 12,264 U.S. adults. The study also found that individuals with excessive iodine intake (urinary iodine concentration ≥ 400 μg/L) had a 19% higher risk of all-cause mortality compared to those with adequate levels (100–299 μg/L) [69]. A 20-year study comparing two Danish communities with different iodine levels in drinking water found that residents of Skagen, where iodine intake was approximately 139 μg/L, had a 40% lower risk of death compared to residents of Randers, where iodine levels were only 2 μg/L. These findings suggest that long-term deficit or excess of iodine intake may be associated with decreased life expectancy [70]. These results are in agreement with our findings.

Notably, this association was observed only in men, suggesting a potential role of sex hormones in iodine-related mortality. Estradiol inhibits NIS expression, reducing the thyroid’s ability to uptake iodine, which may lead to gender differences in the effects of iodine intake [71]. Estrogen increases the level of thyroxine-binding globulin (TBG), a protein that transports thyroid hormones in the blood, which may reduce the amount of free and active T3/T4 hormones despite normal total concentrations [72]. In men, the sensitivity to excess iodine in the context of thyroid disease risk is higher, which may indicate a role of estrogen in the protective effect of iodine in women [73].

Our study has several limitations. First, the sample was drawn from a single institution, and the number of survivors of kidney cancer included in the cohort was relatively small. We have no collected data about environmental or social factors in the patients, thyroid hormone data, dietary, nor information about environmental iodine exposure. However, this is the first study to investigate the impact of serum iodine levels on survival in survivors of kidney cancer. Serum samples were collected at the time of diagnosis, prior to the initiation of treatment. Despite these limitations, our findings provide a valuable foundation for future research and offer an opportunity to collaborate with other investigators globally to validate the role of iodine in improving outcomes for survivors of kidney cancer.

## 5. Conclusions

Both iodine deficiency and excess can have adverse health effects. Our findings indicate that, in survivors of kidney cancer with kidney cancer, serum iodine levels below 63.42 µg/L and above 72.02 µg/L were associated with an increased risk of death, particularly from kidney cancer progression. This information supports researchers and healthcare professionals in several key ways: serum iodine concentration may serve as a prognostic biomarker for kidney cancer progression and mortality. This confirms the need for regular monitoring of iodine levels in kidney cancer survivors to ensure they remain within a safe range, minimizing health risks associated with both deficiency and excess. These findings open the door for future research into iodine modulation as a potential therapeutic target or supportive treatment strategy in kidney cancer care. The results inform the development of clinical guidelines for nutritional and metabolic surveillance as part of survivorship care plans, ensuring that trace elements like iodine are included in comprehensive patient management. The research highlights the importance of balanced iodine intake, which can be translated into dietary recommendations or supplementation strategies tailored to at-risk groups. By identifying critical serum iodine thresholds linked to survival outcomes, this study enables a more nuanced approach to post-cancer care and encourages further research into how trace elements influence cancer progression.

In summary, the discovery of a narrow optimal range for serum iodine in kidney cancer survivors underscores the delicate balance required in micronutrient regulation. It empowers both researchers and practitioners to take more targeted actions—whether in designing studies, formulating care guidelines, or delivering patient-specific interventions—that may ultimately improve survival and quality of life.

## 6. Patents

Based on the results presented in this paper, a patent application has been submitted to the Patent Office of the Republic of Poland (P. 452437).

## Figures and Tables

**Table 1:** Characteristics of the survivors of kidney cancer in the study.

Variables	Overall *n* = 284	Living Individuals *n* = 204	Deceased Individuals *n* = 80
Age of diagnosis (mean)			
≤60 (50.12)	121 (43%)	98 (48%)	23 (29%)
>61 (67.66)	163 (57%)	106 (52%)	57 (71%)
Sex			
Female	118 (42%)	91 (45%)	27 (34%)
Male	166 (58%)	113 (55%)	53 (66%)
Smoking status			
No	95 (33%)	77 (38%)	18 (23%)
Current/Former smoker	189 (67%)	127 (62%)	62 (78%)
Kind of operation			
Nephrectomy	126 (44%)	84 (41%)	42 (53%)
Tumorectomy	158 (56%)	120 (59%)	38 (48%)
Histological features			
* GI	75 (26%)	64 (31%)	11 (14%)
GII	125 (44%)	96 (47%)	29 (36%)
GIII	63 (22%)	39 (19%)	24 (30%)
GIV	21 (7.4%)	5 (2.5%)	16 (20%)
Clear cell carcinoma	245 (86%)	169 (83%)	76 (95%)
Papillary/Chromophobe	39 (14%)	35 (17%)	4 (5.0%)
Death due to cancer			
No	–	–	19 (29%)
Yes	–	–	46 (71%)
Unknown	–	–	15

* GI–GIV—Fuhrman Grade.

**Table 2:** Association between serum iodine concentrations and all-cause mortality among kidney cancer survivors.

	Vital Status			Univariable COX Regression			Multivariable COX Regression *		
Variables	Overall *n* = 284	Alive *n* = 204	Dead *n* = 80	HR ^1^	95% CI	*p*- Value	HR ^1^	95% CI	*p*- Value
II (reference): 63.42–71.96	71 (25%)	61 (30%)	10 (13%)	—	—		—	—	
III: 72.02–81.76	71 (25%)	48 (24%)	23 (29%)	2.63	1.25–5.52	0.011	2.83	1.26–6.34	0.012
IV: 81.85–169	71 (25%)	40 (20%)	31 (39%)	3.68	1.80–7.50	<0.001	2.64	1.19–5.88	0.017

HR ^1^ = Hazard Ratio, CI = Confidence Interval, * multivariate models with covariates including: age, sex, smoking status, type of surgery, histopathological classification and serum levels of selenium, zinc, copper and the zinc-to-copper ratio.

**Table 3:** Survival of kidney cancer survivors according to serum iodine levels among patients who died from kidney cancer.

	Vital Status			Univariable COX Regression			Multivariable COX Regression *		
Variables	Overall *n* = 250	Alive *n* = 204	Dead *n* = 46	HR ^1^	95% CI	*p*-Value	HR ^1^	95% CI	*p*-Value
II (reference): 63.54–71.87	62 (25%)	58 (28%)	4 (8.7%)	—	—		—	—	
I: 34.45–63.51	63 (25%)	57 (28%)	6(13%)	1.55	0.44–5.50	0.5	1.24	0.31–5.06	0.8
III: 71.96–81.67	62 (25%)	48 (24%)	14 (30%)	3.91	1.29–11.9	0.016	4.17	1.14–15.3	0.031
IV: 81.76–169	63 (25%)	41 (20%)	22 (48%)	6.26	2.16–18.2	<0.001	3.94	1.08–14.4	0.038

HR ^1^ = Hazard Ratio, CI = Confidence Interval, * multivariate models with covariates including: age, sex, smoking status, type of surgery, histopathological classification, and serum levels of selenium, zinc, copper, and the zinc-to-copper ratio.

**Table 4:** Kidney cancer–specific survival in men according to serum iodine levels.

	Vital Status			Univariable COX Regression			Multivariable COX Regression *		
Variables	Overall *n* = 140	Alive *n* = 113	Dead *n* = 27	HR ^1^	95% CI	*p*-Value	HR ^1^	95% CI	*p*-Value
II (reference): 62.43–68.79	35 (25%)	34 (30%)	1 (3.7%)	—	—		—	—	
I: 34.45–62.06	35 (25%)	31 (27%)	4 (15%)	4.20	0.47–37.6	0.2	1.06	0.08–14.4	>0.9
III: 68.80–79.94	35 (25%)	28 (25%)	7 (26%)	7.81	0.96–63.5	0.055	3.41	0.31–37.7	0.3
IV: 79.98–169	35 (25%)	20 (18%)	15 (56%)	19.6	2.58–148	0.004	16.5	1.38–196	0.027

HR ^1^ = Hazard Ratio, CI = Confidence Interval, * multivariate models with covariates including: age, sex, smoking status, type of surgery, histopathological classification, and serum levels of selenium, zinc, copper, and the zinc-to-copper ratio.

**Table 5:** Survival of survivors of kidney cancer according to serum I levels among non-kidney-cancer-specific death.

	Vital Status			Univariable COX Regression			Multivariable COX Regression *		
Variables	Overall *n* = 223	Alive *n* = 204	Dead *n* = 19	HR ^1^	95% CI	*p*- Value	HR ^1^	95% CI	*p*- Value
III (reference): 70.02–79.55	56 (25%)	54 (26%)	2 (11%)	—	—		—	—	
I: 0.00–62.86	56 (25%)	50 (25%)	6 (32%)	3.49	0.70–17.3	0.13	2.10	0.38–11.8	0.4
II: 62.91–69.97	55 (25%)	52 (25%)	3 (16%)	1.40	0.23–8.38	0.7	1.35	0.20–8.86	0.8
IV: 79.64–6262.94	56 (25%)	48 (24%)	8 (42%)	3.86	0.81–18.3	0.089	5.41	1.00–29.1	0.050

HR ^1^ = Hazard Ratio, CI = Confidence Interval, * multivariate models with covariates including: age, sex, smoking status, type of surgery, histopathological classification, and serum levels of selenium, zinc, copper, and the zinc-to-copper ratio.

## Data Availability

Our data contain potentially sensitive information; therefore, we have not included them with our manuscript. The Pomeranian University of Medicine Ethics Committee will grant access to all researchers who meet the criteria for access to confidential data.

## Supplementary Materials

The following supporting information can be downloaded at: https://www.mdpi.com/article/10.3390/cancers17213400/s1, Table S1. Survival from kidney cancer depending on serum I levels in women; Table S2. Survival from kidney cancer depending on serum I levels in men; Table S3. Survival of kidney cancer women according to serum I levels among kidney cancer-specific death.
