# Supplementary material for: Serum Iodine Levels and 8-Year Survival in Patients After Kidney Cancer Diagnosis
Source: Cancers (Basel). 2025 Oct 22;17(21):3400. doi: 10.3390/cancers17213400 (PMC12610845; doi:10.3390/cancers17213400)
Supplement: Supplementary file 1 [file cancers-17-03400-s001.zip › cancers-3861366-supplementary.pdf]

Supplementary materials

Table S1. Survival from kidney cancer depending on serum I levels in women.

	Vital status			Univariable COX Regression			Multivariable COX Regression		
**Variables**	**Overall** n **= 118**[1]	**Alive** n **= 91**[1]	**Dead** n **= 27**[1]	**HR**[2]	**95% CI**	p**-value**	**HR**[2]	**95% CI**	p**-value**
I (reference): 0.00 – 66.32	30 (25%)	25 (27%)	5 (19%)	—	—		—	—	
II: 66.66 – 74.76	29 (25%)	24 (26%)	5 (19%)	1.00	0.29 – 3.45	> 0.9	0.64	0.14 – 2.85	0.6
III: 74.83 – 83.36	29 (25%)	23 (25%)	6 (22%)	1.26	0.38 – 4.13	0.7	0.46	0.10 – 2.00	0.3
IV: 83.47 – 179.26	30 (25%)	19 (21%)	11 (41%)	2.26	0.79 – 6.51	0.13	0.57	0.15 – 2.22	0.4

[1]n (%), [2]HR = Hazard Ratio, CI = Confidence Interval.

Table S2. Survival from kidney cancer depending on serum I levels in men.

	Vital status			Univariable COX Regression			Multivariable COX Regression		
**Variables**	**Overall** n **= 166**[1]	**Alive** n **= 113**[1]	**Dead** n **= 53**[1]	**HR**[2]	**95% CI**	p**-value**	**HR**[2]	**95% CI**	p**-value**
II (reference): 62.43 – 68.80	41 (25%)	35 (31%)	6 (11%)	—	—		—	—	
I: 34.45 – 62.06	42 (25%)	31 (27%)	11 (21%)	2.00	0.74 – 5.41	0.2	0.77	0.24 – 2.47	0.7
III: 69.14 – 79.94	41 (25%)	27 (24%)	14 (26%)	2.68	1.03 – 7.00	0.043	1.40	0.47 – 4.15	0.5
IV: 79.98 – 1,563,016.38	42 (25%)	20 (18%)	22 (42%)	4.33	1.75 – 10.7	0.002	2.63	0.85 – 8.14	0.094

[1]n (%), [2]HR = Hazard Ratio, CI = Confidence Interval.

Table S3. Survival of kidney cancer women according to serum I levels among kidney cancer-specific death.

	Vital status			Univariable COX Regression			Multivariable COX Regression		
**Variables**	**Overall** *n* **= 110**[1]	**Alive** *n* **= 91**[1]	**Dead** *n* **= 19**[1]	**HR**[2]	**95% CI**	***p*-value**	**HR**[2]	**95% CI**	***p*-value**
I (reference): 49.50 – 66.32	28 (25%)	25 (27%)	3 (16%)	—	—		—	—	
II: 66.66 – 74.76	27 (25%)	24 (26%)	3 (16%)	0.97	0.19 – 4.79	> 0.9	0.77	0.09 – 6.55	0.8
III: 74.83 – 82.76	27 (25%)	22 (24%)	5 (26%)	1.75	0.42 – 7.32	0.4	0.36	0.03 – 4.73	0.4
IV: 83.36 – 179.26	28 (25%)	20 (22%)	8 (42%)	2.67	0.71 – 10.1	0.15	0.30	0.05 – 1.88	0.2

[1]n (%), [2]HR = Hazard Ratio, CI = Confidence Interval.
